# Expected vs. Actual Refractive Error in Patients Presenting With Phacomorphic Glaucoma

**DOI:** 10.7759/cureus.18076

**Published:** 2021-09-18

**Authors:** Alisha Khambati, Jahan Tajran, Sarah Syeda, Arif Musa, Vaama Patel, Justin Tannir

**Affiliations:** 1 School of Medicine, Wayne State University, Detroit, USA; 2 Ophthalmology, University of Texas Southwestern Medical Center, Dallas, USA; 3 Kresge Eye Institute, Wayne State University, Detroit, USA

**Keywords:** phacomorphic glaucoma, retrospective study, refractive error, spherical equivalence, cataract surgery

## Abstract

Aim

Phacomorphic glaucoma (PG) is a rare but clinically significant presentation requiring emergent cataract surgery. We chose to investigate whether the expected refractive error based on the intraocular lens (IOL) calculations differed from the expected refractive outcome post-surgery.

Materials & Methods

A retrospective analysis of patients with PG between 2009 to 2018 who underwent cataract surgery and had postoperative refraction was included. Information collected included presenting and postoperative best-corrected visual acuity (BCVA), intraocular pressure (IOP) pre­ and postoperatively, and the presence of corneal edema. Predicted spherical equivalence (SphEq) data was collected from IOL calculations, and postoperative SphEq was calculated from postoperative manifest refraction.

Results

Twenty patients with PG who underwent cataract surgery were identified; of these, 10 patients and 10 eyes who underwent manifest refraction post­op were included. Mean BCVA at presentation was 20/544 [Logarithm of Minimal Angle of Resolution (LogMAR) 1.44], and mean pre-op IOP was elevated at 24.6 ± 14.2 mmHg. Mean BCVA measured at one month post-op improved to 20/192 (LogMAR 0.983). Mean IOP decreased to 19 ± 8.8 mmHg at one month post-op. The mean difference between the predicted and actual refractive error, as calculated by SphEq was ­0.088 (p=0.570).

Conclusion

The study shows an improvement in visual acuity and IOP post-cataract surgery in patients with PG, as would be expected. The study also demonstrates that patients ended up with a slightly more myopic refractive error than expected postoperatively, and illustrates the clinical variability in postoperative refractive outcomes from a large standard deviation. This is a new and clinically important finding, although not statistically significant, that has not been previously published.

## Introduction

Glaucoma is the second leading cause of blindness worldwide and has many forms [1]. In adults between the ages of 40 and 80, the prevalence of glaucoma is as high as 3.54% [2]. Phacomorphic glaucoma is a rare but clinically significant subtype of secondary angle-closure glaucoma that occurs due to a hyper mature cataract [3]. The enlarged lens of the hyper mature cataract displaces the iris forward and into apposition with the conventional and uveoscleral aqueous drainage pathways, creating a secondary angle-closure [1]. Pupillary block is also a well-known secondary effect of lens intumescence, and can further limit aqueous outflow [3]. The result is a rapidly increased intraocular pressure (IOP) and, consequently, closed or narrow-angle glaucoma [4-6]. Moreover, increased IOP has the potential to induce vision loss secondary to damage of the optic nerve [4,6].

Therefore, the treatment of phacomorphic glaucoma requires decreasing IOP through pharmacotherapy or surgical intervention. Initially, the IOP is reduced to a stable level with either topical glaucoma medications or oral acetazolamide [7]. This is followed by the mainstay of definitive treatment: cataract surgery [5]. Given that cataracts account for 41.8% of blindness worldwide, early intervention with cataract surgery has the potential to not only significantly reduce the global burden of blindness but also decrease the prevalence of phacomorphic and other lens-related glaucomas [8].

Due to the potential for complications of increased IOP associated with phacomorphic glaucoma, cataract surgery constitutes a high-risk and challenging procedure [5]. Complications of cataract surgery include weakened zonules tearing and expulsive hemorrhaging [9]. Ultimately, effective extraction of the cataract and implantation of an intraocular lens (IOL) without unintended sequelae requires accurate preoperative measurement [10]. In phacomorphic glaucoma, the presence of corneal edema and a hyper mature cataract can impede the view, making it difficult to calculate accurate IOL. As a result, this study investigates a method of calculating more accurate IOL in phacomorphic glaucoma by comparing the expected refractive error pre-cataract surgery to the expected refractive outcome post-cataract surgery.

## Materials and methods

Following Wayne State University Human Research Protection Program Institutional Review Board approval (IRB: 104517MP4X), a retrospective case series analysis of the electronic medical records (EMR) was conducted for patients with a formal diagnosis of phacomorphic glaucoma between 2009 to 2018 who had undergone cataract surgery. All patients received cataract surgery at a single medical center in the United States. Patient confidentiality was maintained in compliance with the US Health Insurance Portability and Accountability Act and the Declaration of Helsinki for human subjects.

A total of 20 patients with phacomorphic glaucoma that underwent cataract surgery were initially identified. The EMR information was cross-referenced with the patient operative reports. All patients exhibited signs and symptoms of secondary angle-closure glaucoma. A total of 10 patients and 10 eyes had undergone manifest refraction, between two weeks to 90 days post-cataract surgery. Also, the varied range in postoperative manifest refraction was performed to account for the resolution of short-term postoperative challenges like corneal and macular edema. Patients with acute primary angle-closure glaucoma diagnosis were excluded. 

For each patient, preoperative IOP, age of presentation, sex, race, best-corrected visual acuity (BCVA) pre- and post-cataract surgery, past ocular history and surgery, presence of diabetes, and corneal edema were collected over a nine-year period. The type of cataract surgery performed (posterior chamber intraocular lens (PCIOL) or anterior chamber intraocular lens (ACIOL)) and the eye (OD or OS) in which the cataract extraction was done were also collected. Data collection was performed consistently using a standardized data abstraction sheet with inclusion and exclusion criteria established a priori. Postoperative medical records were dated to the most recent manifest refraction after cataract surgery.

IOL calculations obtained prior to cataract surgery constituted the predicted spherical equivalence (SphEq) data. The Zeiss IOLMaster 500 (Zeiss, Jena, Germany) [11] was the diagnostic equipment used to make preoperative measurements. Postoperative manifest refraction was used to calculate the post-cataract extraction spherical equivalence. For each patient, the difference between predicted spherical equivalence preoperatively and actual spherical equivalence postoperatively was taken and then averaged with the predicted and actual spherical equivalence differences for a total of the 10 included patients to obtain the refractive error. 

Spherical equivalence and visual acuity calculations

Prior to cataract surgery, the predicted spherical equivalence calculations and all other IOL calculations were done preoperatively, using the Holladay 1 equation [12]. Post-cataract surgery, the spherical equivalence was calculated with a specific refraction formula. In this formula, Spherical Equivalence = Postoperative sphere power (in diopters) + ½ * Postoperative cylinder power (in diopters). In addition, pre and postoperative BCVA was converted into Logarithm of Minimal Angle of Resolution (LogMAR) units through an online Snellen to LogMAR Visual Acuity conversion calculator [13].

Statistical analysis

Descriptive statistics were employed to quantify patient data. A Snellen chart [14] was used for its utility in BCVA measurements in pre- and post-cataract surgery. For statistical analysis, the Snellen chart values were converted into LogMAR units. The data were analyzed and converted into figures, graphs, and means using Microsoft® Excel Version 15.11.2 (Microsoft, Redmond, WA, USA). A paired t-test and Wilcoxon signed-rank test (STATVIEW 5.0.1 software (SAS Institute, Cary North Carolina, USA) was used to compare the mean differences between the predicted and actual SphEq values. A p-value less than 0.05 was considered to be statistically significant.

## Results

Of 20 patients presenting with phacomorphic glaucoma that underwent cataract surgery between 2009 and 2018, 10 patients and 10 eyes with postoperative manifest refraction were included. The mean presenting age was 75.7 years (range, 58-97 years), and 90% of the patients were African American. The female to male ratio was approximately 2.3:1 (70% were female and 30% were male). Three out of the ten included patients with phacomorphic glaucoma were diabetic, while seven out of the ten had a past ocular history, of which three underwent a previous ocular surgery.

Also, three patients presented with corneal edema, and two patients had undergone a peripheral iridotomy at the time of IOL calculations prior to cataract surgery. Postoperatively, four patients presented with corneal edema, two of which had persistent corneal edema that presented prior to cataract surgery. Overall, nine patients underwent a PCIOL, while only one patient had an ACIOL for the type of cataract surgery performed.

**Figure 1:**
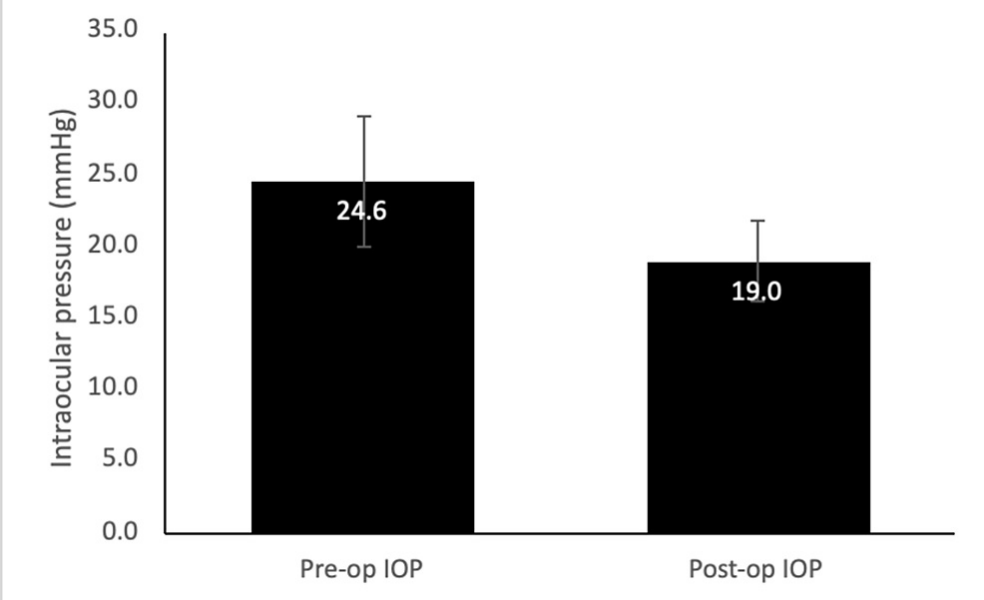
Mean and standard deviation for intraocular pressure before and after surgery are reported.

The mean BCVA of presenting phacomorphic glaucoma patients’ pre-cataract surgery was LogMAR 1.44 ± 1.19 ( Snellen mean, 20/544; Snellen range, 20/30 to Light Perception). The mean postoperative BCVA improved to 0.98 ± 1.01 ( Snellen mean, 20/192; Snellen range, 20/20 to Hand Motion). The mean preoperative IOP was 24.6 ± 14.2 mmHg (range, 12-50 mmHg). The mean postoperative IOP decreased to 19.0 ± 8.8 mmHg (range, 12-40 mmHg). A summary of the pre- and post-cataract surgery IOP and BCVA is displayed in Figure 1 and Table 1, respectively.

**Table 1:** Best-Corrected Visual Acuity (BCVA) are provided for before and after surgery. Snellen visual acuity (SVA) means and ranges are listed.

Table 1. Best-Corrected Visual Acuity Before and After Cataract Surgery		
Snellen Visual Acuity	Preoperative	Postoperative
Mean	20/544	20/192
Range	20/30^a^	20/20^b^
^a^LM: light perception ^b^HM: hand movement		

**Figure 2:**
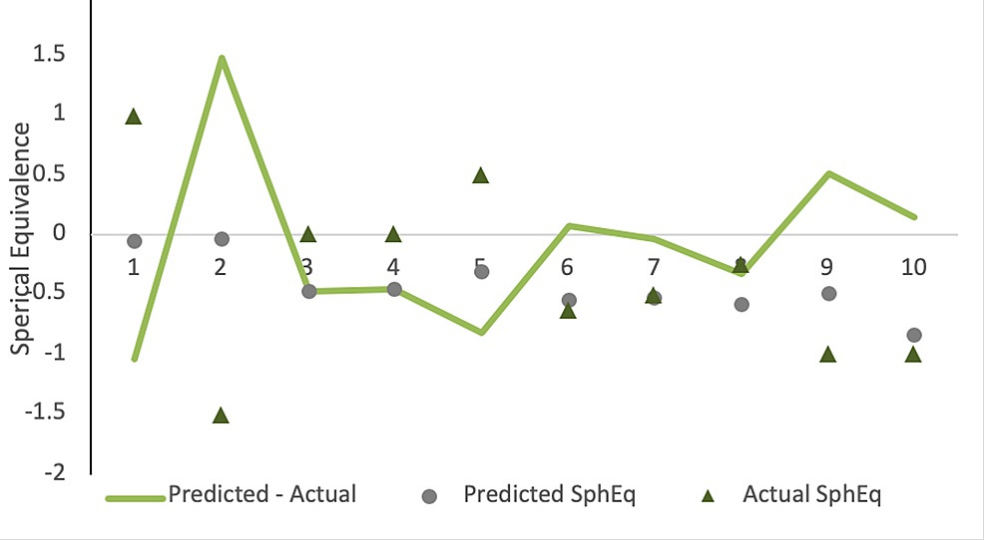
The expected refractive error, actual refractive error, and difference between refractive and actual errors are reported.

The mean difference between the expected and actual refractive error, which was generated through the SphEq, was -0.088 ± 0.72 (p=0.570). The expected vs. actual refractive error is depicted in Figure 2.

## Discussion

Phacomorphic glaucoma has a sudden onset progression that starts with a hyper mature cataract, creating angle crowding and possibly pupillary blockage [3]. Notably, there is a high prevalence of the disease in developing countries [5], Asian countries, and the elderly populations [4]. In addition, patients over the age of 60, with a shallow anterior chamber and an axial length of over 23.7 mm, are at increased risk for phacomorphic angle closure [15,16]. As a result, it has been recommended that patients with these risk factors should be treated immediately to prevent irreversible vision loss [3]. However, cataract surgery may be difficult to perform in patients with phacomorphic glaucoma. Corneal edema and the intumescent cataract itself make intraocular lens (IOL) calculations challenging, leading to unpredictable visual outcomes post-surgery [5,9].

Although some patients postoperatively possess a hyperopic refractive error individually (n=2), most patients ended up with a myopic refractive error difference (n=8). The difference in refractive error outcomes pre- and post-cataract surgery was measured, generating a mean difference of -0.088 ± 0.72 (p=0.570) based on spherical equivalence values. Although this value reveals a postoperative myopic refractive error mean difference compared to the IOL calculations prior to cataract surgery, the most compelling outcome is the variability in the postoperative refractive outcomes seen with our large standard deviation. This is a novel finding that has not yet been published in the literature and warrants further study in a larger cohort. 

The observed fluctuations in myopic and hyperopic refractive errors may be due to several reasons. One explanation is that a multitude of different physiological factors such as the patient's hyper mature cataract, possible corneal edema, or history of peripheral iridotomy may affect the accuracy of the IOL calculations for the lens implant. Alternatively, the fluctuations may be attributed to the small sample size, which is indicative of how uncommon cases of phacomorphic glaucoma are [17]. Despite data collection from a large, metropolitan institution over a 10-year period, only 10 patients and 10 eyes were eligible for inclusion in this retrospective case series. Although 20 patients were identified in the electronic medical records as diagnosed with phacomorphic glaucoma from 2009 to 2018, 10 patients were ultimately lost to postoperative follow-up. While it is not known why these patients did not return, studies suggest that loss to follow-up is more common in urban settings [18].

In addition to the fact that there were only 10 patients with 10 eyes used in this study, a larger standard deviation in postoperative refractive outcomes was expected in phacomorphic glaucoma patients than in traditional cataract patients. This is due to the fact that surgery on patients with phacomorphic glaucoma bears a higher risk [1]. Furthermore, dilation of the pupil prior to surgery may exacerbate the potential for additional angle-closure glaucoma, reducing the predictive accuracy of the IOL implant and worsening the refractive error [1]. Additionally, the variability in postoperative spherical equivalence, across 20/20 to hand motion visual acuities, suggests a possible consequence from the low power of the study and warrants further investigation. 

We calculated an 80% decrease in IOP postoperatively compared to pre-cataract surgery, which is supported by the literature [3]. Initially, 30% of patients had corneal edema prior to surgery. After cataract extraction, 40% of patients possessed corneal edema, a common complication observed in the immediate postoperative period. The mean preoperative BCVA was LogMAR 1.44 ± 1.19 and improved to a postoperative mean BCVA of 0.98 ± 1.01, in which the range of patients saw 20/20 to Hand Motion. Thus, in 70% of the patients, there was an improvement in BCVA, while 20% of the patients had a visual acuity that remained the same pre and postoperatively. In agreement with other studies in the literature, functional visual acuity outcomes remain favorable following phacomorphic glaucoma surgery despite postoperative corneal edema [3] and despite the fact that the study did not show a significant difference in pre and postoperative refractive error (p=0.570). 

The insignificant difference between the pre and postoperative refractive errors prompts future prospective studies in order to verify these findings in larger sample sizes. In addition, future studies should aim to elucidate the mechanism behind myopic refractive error following cataract extraction for phacomorphic glaucoma. If these findings are confirmed, additional studies should investigate whether a two-stage surgery, where the cataract would be extracted to control the increased intraocular pressure and then delayed intraocular lens implantation in a controlled environment, would help control the variability in postoperative refractive outcomes.

## Conclusions

The study demonstrates a reduction in IOP and an improvement in visual acuity outcomes post-cataract surgery in patients presenting with phacomorphic glaucoma, in accordance with the hypothesis. Although the majority of the patients obtained a more myopic refractive error at -0.088 ± 0.72 (p=0.570) after cataract extraction, there was clinical variability in the postoperative refractive outcomes, shown by the lack of statistical significance and the large standard deviation. This myopic refractive error finding is novel clinically, as it can impact a patient's visual acuity, and has not been previously described in the literature. Since the study is underpowered, future studies with larger sample sizes should be done to confirm this study’s findings and investigate the methods to the myopic refractive error. If this is indeed the case, it will be important to look into the possible staging of the procedure, with cataract extraction followed by a separate IOL implantation, to see if it impacts the standard deviation in postoperative refractive outcomes and the refractive error.
